# Preventive and Social Medicine

**Published:** 2008-10

**Authors:** Jarnail S Thakur

**Affiliations:** Associate Professor, PGIMER School of Public Health, Chandigarh. E-mail: jsthakur64@gmail.com

It is a book on multiple choice questions (MCQ) in Preventive and Social Medicine. All relevant topics have been covered meticulously including current developments and evolution of Preventive Medicine. It would serve as an excellent resource material for those preparing for entrance examinations. The presentation of this book is different from other MCQ book. Authentic explanation justifying the correct answer is really a unique feature which will help the readers to understand the topic rather just cramming it. The book is likely to serve as a benchmark on MCQ in Preventive and Social Medicine

